# Implementation, Processes and Outcomes of Advance Care Planning: A Culturally and Contextually Appropriate Programme Theory Developed in Chinese Long‐Term Care Facilities

**DOI:** 10.1111/hex.70291

**Published:** 2025-05-08

**Authors:** Yuxin Zhou, Ariel Wang, Clare Ellis‐Smith, Debbie Braybrook, Haixia Feng, Richard Harding

**Affiliations:** ^1^ Cicely Saunders Institute of Palliative Care, Policy and Rehabilitation, Florence Nightingale Faculty of Nursing Midwifery & Palliative Care King's College London London UK; ^2^ Nuffield Department of Primary Care Health Sciences University of Oxford Oxford UK; ^3^ Department of Nursing, Affiliated ZhongDa Hospital, School of Medicine Southeast University Nanjing China

**Keywords:** advance care planning, end‐of‐life care, long‐term care facilities, older people, palliative care, Theory of Change

## Abstract

**Background:**

Despite advance care planning (ACP) being associated with positive outcomes for residents in long‐term care facilities (LTCFs), the causal pathways between ACP and these outcomes are context‐specific and less understood. This lack of clarity can hinder the cultural adaptation and evaluation of ACP interventions. This study aimed to develop a programme theory that outlines the causal pathways through which the ACP is hypothesised to achieve impacts in Chinese LTCFs, with a focus on understanding its implementation, processes and outcomes.

**Methods:**

Exploratory qualitative design incorporating Theory of Change (ToC) methodology. Two ToC workshops (one face‐to‐face and one online) were held with 37 participants experienced in caring for residents or older people. The process was informed by a realist review and primary qualitative study. A programme theory was developed through thematic analysis, generating a ToC map depicting implementation, processes and outcomes of ACP in LTCFs.

**Results:**

The programme theory was constructed to outline the causal pathways of ACP in LTCFs, populating five ‘precondition’ domains: (1) buy‐in from government and facility leadership, (2) availability of external and internal resource, (3) adequate training and awareness for public and facility, (4) identification of residents who are ready for ACP and (5) culturally sensitive communication. Nine intervention components were identified that target preconditions, such as raising ACP awareness and providing staff training and mentoring. The potential impacts of ACP were identified, for example, fostering public attitudes towards a ‘good death’ and increasing public awareness and acceptance of palliative care.

**Conclusions:**

Our mid‐range programme theory can serve as a heuristic tool, adaptable for context‐specific ACP interventions in other countries, enhancing the likelihood of achieving intended impacts. In particular, intervention components focused on family involvement can be transferable to East Asian regions, where relational autonomy and family‐centred decision‐making are emphasised. The programme theory is ready for feasibility testing for residents in Chinese LTCFs.

**Patient or Public Contributions:**

We were guided by patient and public involvement members including two residents and one family member of a resident throughout the study. They supported the overall development of programme theory, including reviewing the theory and interpreting findings.

## 1 Introduction

By 2050, the global population aged 65 and above is projected to reach 1.6 billion [1]. In China, the number of adults over 65 is expected to reach 370 million, accounting for 22.9% of the world's ageing population [2]. This demographic shift may leave 16 million older people without sufficient informal care due to factors such as declining fertility, the one‐child policy which has affected family structures, and rural–urban migration [3]. Long‐term care facilities (LTCFs), which offer 24‐h aged care, are increasingly the primary setting for older people who lack informal care and have cognitive and functional impairments [3]. These individuals live with multimorbidity and high dependency and experience high physical symptom burden and psychosocial needs [4]. Consequently, many may lose decision‐making capacity about their care and experience difficulties in expressing their wishes and concerns [5].

Advance care planning (ACP) is a voluntary process designed to support individuals in having important discussions with family and care providers about their values, goals and preferences for future treatment and care before they lose capacity [6]. Systematic reviews have demonstrated the effectiveness of ACP in LTCFs such as increased documentation of end‐of‐life care preferences and decreased hospitalisation rates [5, 7]. However, the actual implementation of ACP within LTCFs has been inadequate. Research suggested that residents and their families have minimal experience with ACP conversations [8]. A recent study in Swedish nursing homes showed that while 97% of residents had documented care plans, the prevalence of ACP discussions remained unclear [9]. Additionally, there is a notable lack of ‘systems thinking’ that incorporates implementation considerations beyond the collective level and integrates multiple intervention components [10].

ACP in mainland China gained attention relatively late, in the early 21st century, and remains in its infancy [11]. Efforts to promote ACP in mainland China have largely been tied to Living Wills, that is, legal documents outlining what life‐sustaining treatments a person does or does not want at the end of life [12]. However, the concept of ACP is still largely unknown to the public and healthcare professionals, and there is a lack of localised programmes [13, 14]. A major challenge in expanding access to ACP globally is that most of the evidence on its effectiveness and implementation originates from Western contexts [15]. Several reviews have highlighted this fact and that cultural differences play a crucial role in its successful implementation [15, 16, 17]. ACP utilising a Western framework emphasises the importance of individualism and patient autonomy. However, in Asian countries, particularly those influenced by Confucianism and collectivism, such as China, Japan and Korea, relational autonomy (i.e., the individual within a socially embedded network) and family‐centred decision‐making are preferred [18]. There are significant cultural barriers to the implementation of ACP in Chinese LTCFs, including a lack of discourse on end‐of‐life care, relational decision‐making processes, low awareness of palliative care, and distrust between residents/family and healthcare professionals [14]. It is essential to develop ACP interventions rooted in the Chinese cultural and ethical context, aligning with the core theoretical foundation of Western frameworks that emphasise respect for individual wishes and preferences [14, 19].

ACP is a complex intervention with multiple interacting components that require adaptation to local contexts [6, 7]. A deeper understanding of the programme theory (i.e., how and why a programme or intervention works) will enable the development of ACP interventions which can be implemented successfully in a local context to achieve desired impacts [20]. However, a clear and detailed process for rigorously translating foundational work into complex interventions and their underlying programme theory remains limited [21]. Consequently, the effective replication and adaptation of context‐specific ACP interventions remain poorly understood. Theory of Change (ToC) is a theory‐driven approach that aids in developing programme theory by clarifying the processes of change within interventions which achieve impacts [22]. Such a programme theory is developed specifically for a given intervention, drawing on existing evidence and involving stakeholders [22]. ToC has been used previously to develop the ACP programme theory in Belgian nursing homes [20]. However, there is still a lack of evidence on culturally and contextually appropriate programme theory supporting ACP in LTCFs in non‐Western countries. This study aimed to develop a programme theory that outlines the causal pathways through which the ACP is hypothesised to achieve impacts in Chinese LTCFs, with a focus on understanding its implementation, processes and outcomes.

## 2 Methods

### 2.1 Study Design and Theoretical Underpinnings

An exploratory qualitative design incorporating the ToC methodology was used to explore the implementation, processes and outcomes of ACP. This study is part of a sequential multi‐methods project to develop and evaluate a contextually and culturally appropriate ACP programme for Chinese LTCFs. The study design followed the Medical Research Council (MRC) framework for complex interventions [23] and was informed by the findings of our realist review [24] and qualitative study [14]. The study reporting followed the checklist for reporting ToC [25] and the Consolidated criteria for Reporting Qualitative research checklist [26] (see Supporting Information 1).

**Figure 1:**
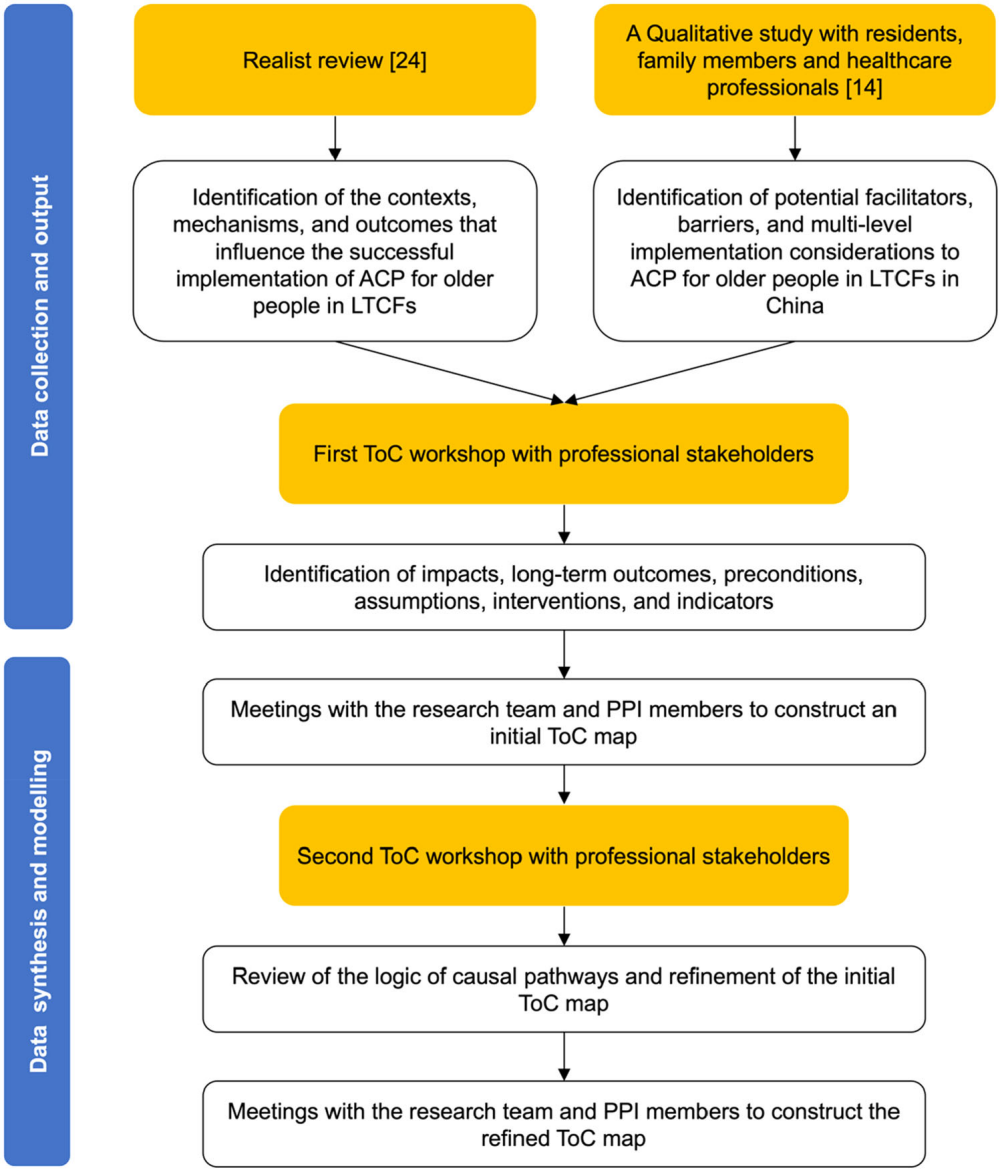
The methods and process for developing and modelling the programme theory in this study, drawing on methodology from Nooijer et al. (2021) [28] and Gilissen et al. (2018) [20]. ACP, advance care planning; LTCFs, long‐term care facilities; PPI, patient and public involvement; ToC, theory of change.

ToC is ‘a theory of how and why an initiative works’ and is recommended for use in complex intervention development to enhance stakeholder engagement and tailor interventions to their local context (see Supporting Information 2 for terminology) [22]. It outlines the local assumptions necessary for successful implementation, specifies the key intervention components required to achieve intended outcomes and identifies relevant indicators to measure success [25]. These elements are integrated and displayed on a ToC map, which is a graphic depiction of the causal pathways through which an intervention is expected to achieve its impact [22]. In this study, we adopted the workshop format proposed by Breuer et al. (2014) [27] and used ToC to develop the programme theory, which is visually represented in a ToC map. The methods and process for developing and modelling the programme theory in this study were drawn from previous studies [20, 28] and are described in Figure 1.

### 2.2 Participants and Recruitment

There are three types of LTCFs in mainland China: public social welfare facilities, nursing homes and residential care facilities. These can be broadly categorised into three ownerships: government‐built and government‐operated, government‐built and privately‐operated, and privately‐built and privately‐operated [3]. The last category accounts for more than 70%, making up the majority of current LTCFs in China [29]. By the end of 2021, there were 39,961 registered LTCFs for older people in China, with 5.036 million beds [30].

Participants comprised professional stakeholders from China with various roles, including clinical managers, health and social care professionals, palliative care specialists and academics. Eligible participants were those working in three types of LTCFs with experience in caring for older people or those working in relevant non‐governmental organisations (NGOs), hospitals or universities who were involved in the care of older people. Participants were recruited using purposive sampling, with criteria including age, gender, professional role, professional title and employer to ensure sample diversity and facilitate a broader understanding of different perspectives. A sample size of up to 40 participants across the workshops was planned, guided by the concept of information power [31] and insights from a workshop study [32].

We identified potential participants based on the research team's clinical and academic networks and used snowballing methods. These individuals were invited via emails containing participant information sheets and received follow‐up phone calls. Written informed consent was provided via email or in person before the workshops. Recruitment of participants for the two workshops was conducted separately, and participants were not required to attend both.

### 2.3 ToC Workshops: Structure and Content

Two workshops were conducted between September 2022 and January 2023. The in‐person workshop took place in Nanjing city, Jiangsu Province, and the online workshop via VooV meeting. The workshops were led by a female palliative care PhD student (Y.Z.), who had received ToC methods training, and supported by master's level nursing students who took notes. Before the workshops, the agenda and demographic questionnaire, which included questions such as work experience, professional roles and employers, were emailed to participants. All workshops followed a similar process. First, a presentation was given on the study background, the realist review [24] and qualitative study findings [14], and an explanation of the ToC approach. Secondly, a small group discussion focused on questions informed by the previous study [20] and ToC workshop guidance [33] (see Table 1). Thirdly, group representatives summarised the feedback to the plenary for broader discussion. Lastly, groups reached consensus and summarised the key messages. Participants were assigned to parallel small groups based on their professional roles and employer to ensure a variety of roles in each group. To maintain consistency within and across groups, group representatives identified priorities and reached a consensus before sharing feedback with the plenary. The facilitator reviewed the logic of the ToC map with the plenary to ensure agreement before concluding the workshops. Workshops were audio‐ or video‐recorded, with event artefacts (see Supporting Information 3) including discussion notes, sticky notes, photographs, online chat messages and post‐workshop debrief comments also treated as data for analysis [34].

**Table 1:** Discussion topics and questions of two ToC workshops.

Discussion topics	Discussion questions
Workshop 1: Identifying key elements and mapping the programme theory	
Identify assumptions	(1) Why were the residents' wishes or needs unmet in the vignette? (2) What challenges and opportunities may we encounter when delivering ACP in LTCFs?
Agree on the impact	What should be the ultimate goal of the ACP in LTCFs?
Identify long‐term outcomes and preconditions	(1) What long‐term outcomes are required to achieve the impact? (2) What necessary preconditions (e.g., buy‐in, resources, training and awareness, identification, and communication) are needed to reach these long‐term outcomes?
Identify intervention components	(1) What key intervention components are required to achieve preconditions and long‐term outcomes? (2) Who, what, when, where and how should these interventions be implemented?
Identify indicators of success	(1) How can we measure the successful implementation of ACP? (2) Who or what will be impacted; how does the indicator have to change and how long will it take to bring about change?
Logic and quality check	Is the draft ToC map reasonable, flexible and testable?
Workshop 2: Reviewing the logic of causal pathways and refining the programme theory	
Review and refine the ToC map	Are there any key elements (i.e., impact, long‐term outcomes, preconditions, intervention components, assumptions and indicators) missing from the map?
Logic and quality check	Is the refined ToC map reasonable, flexible and testable?

Abbreviations: ACP, advance care planning; LTCFs, long‐term care facilities; ToC, Theory of Change.

#### 2.3.1 Workshop 1: Identifying Key Elements and Mapping the Programme Theory

The first in‐person workshop aimed to identify key elements of the ToC and develop a visual representation (ToC map) of causal pathways. The discussion began with challenges and assumptions of ACP in LTCFs, followed by an agreement on and prioritisation of the impacts and long‐term outcomes. This was achieved by participants reflecting on the logic model developed in the realist review [24] and a vignette adapted from a case in the qualitative study [14]. Afterwards, participants worked backwards, identifying preconditions required to achieve long‐term outcomes, intervention components and indicators. Participants (health and social care professionals and clinical managers) were mixed across five tables, with five members at each table. Using physical sticky notes, the facilitator (Y.Z.) documented the participants' feedback on A1 worksheets labelled with the key ToC terms (i.e., impact, long‐term outcome, precondition, assumption, intervention and indicator) [22] and co‐constructed an initial ToC map. After the first workshop, the hand‐drawn ToC map was converted into an electronic format.

#### 2.3.2 Workshop 2: Reviewing the Logic of Causal Pathways and Refining the Programme Theory

The second online workshop aimed to review the logic of causal pathways and refine the programme theory. The initial ToC map was presented for feedback on the elements and relationships depicted and to assess logical plausibility. Participants (health and social care professionals, clinical managers and academics) were mixed and divided into three breakout rooms, with five participants in each room accompanied by two research team members (a facilitator and a scribe). The scribe recorded group comments in real time on the pre‐created online discussion board Padlet. Subsequently, Y.Z. led a whole group discussion and co‐constructed the ToC map with participants virtually using Lucidchart, a web‐based diagramming application. Following the workshop, a refined ToC map was developed to visually represent the programme theory of ACP in Chinese LTCFs.

### 2.4 Data analysis

Audio and video recordings were transcribed verbatim and manually checked for accuracy by Y.Z. Y.Z. conducted codebook thematic analysis using NVivo 12 (QSR International (UK) Ltd). A coding scheme was pre‐developed based on the key elements of the ToC [22], that is, impact, long‐term outcome, preconditions (buy‐in, resources, training and awareness, identification and communication), assumption, intervention and indicator, and informed by the implementation logic model of ACP in LTCFs developed in the realist review [24]. Data was deductively coded and categorised into themes and sub‐themes. The themes and sub‐themes were subsequently mapped onto the ToC maps. All themes/sub‐themes, key data extracts and ToC maps were checked by A.W. (a female bilingual researcher with experience in qualitative research) and reviewed by the research team (H.F., C.E.S., D.B. and R.H.).

Complex interventions are better understood by considering the dynamics of context and system, as well as the systems that the interventions set out to change [23]. The Social Ecological Model facilitates a comprehensive ‘whole systems strategic approach’ necessary for effective ACP implementation [35]. Based on the Social Ecological Model [36], the preconditions, long‐term outcomes and impacts that underpin the causal pathways were mapped to system, organisational, interpersonal and individual levels.

To ensure the rigour and reflexivity of data analysis, we adopted the quality criteria outlined in Table 2, drawing on a ToC workshop study [34] and literature on rigour in qualitative research [37, 38, 39].

**Table 2:** Adopted quality criteria in this study.

Quality criteria	How it was fulfilled in this study
Rich rigour (sample, contexts and data in the field)	We collected data from 37 participants, including clinical managers, health and social care professionals, palliative care specialists and academics with diverse backgrounds (e.g., social work, nursing, clinical medicine, sociology, ethics and palliative care) and worked across different LTCFs or relevant care settings. To facilitate analysis, multiple data sources were used, including audio and video recordings, discussion notes, sticky notes, photographs, online chat messages and post‐workshop debrief comments.
Sincerity (reflexivity and transparency)	The data collection and data analysis processes were outlined transparently. Reflexivity was embedded throughout the data analysis process, involving researcher team members' personal experiences, prior knowledge, assumptions and their own biases on the research topic, participants and culture. We also engaged ‘critical friends’ in the data analysis processes [37]. After each workshop, Y.Z. had critical dialogues with PPI members and the research team regularly to encourage reflexivity, manage convergence and divergence, and develop data interpretations.
Credibility (thick description and triangulation)	The research team comprised members with diverse cultural backgrounds and specialities (e.g., nursing, social work, occupational therapy, gerontology and palliative care). We adopted an iterative and collaborative approach to translation and data analysis, as used in our primary qualitative study [14]. This approach allowed team members to explore data from different perspectives, enhancing conceptual understanding.
Resonance (generalisation and transferability)	The data analysis was grounded in Chinese sociocultural, drawing on ToC and Social Ecological Model with a diverse sample. This enhances the transferability of the findings to similar contexts and maximises the potential for readers to reflect on their applicability within their settings.
Meaningful coherence	The logic of causal pathways in the ToC map was informed based on evidence from our realist review [24], qualitative study [14], inputs from ToC workshops and comparisons with existing ToC from other studies [20, 40].

Abbreviations: LTCFs: long‐term care facilities; PPI: patient and public involvement; ToC: Theory of Change.

### 2.5 PPI

Two residents in LTCFs and one family member of a resident were consulted as patient and public involvement (PPI) members to ensure the views of service users were incorporated. They reviewed the ToC maps, provided feedback on the emerging theory, guided interpretation of findings and checked the relevance of the interventions to them.

### 2.6 Ethical Approval

This study was approved by King's College London Research Ethics Committee (Ref: HR/DP‐20/21‐23549) and the Independent Ethics Committee for Clinical Research of Zhongda Hospital, Affiliated to Southeast University (Ref: 2021ZDSYLL277‐P01).

## 3 Results

### 3.1 Characteristics of Participants

The in‐person and online workshops lasted for 5 and 2.5 h, respectively. Of the 45 potential participants who were approached, five declined to take part in the study due to being busy with work (*n* = 3) or personal affairs (*n* = 2). In total, 37 professional stakeholders participated, with 25 in the first workshop and 15 in the second. Three participants attended both. The sample consisted of 18 health and social care professionals, 13 clinical managers and 6 academics (see Table 3). Their mean age was 35 years, with the majority (78.4%) being female. Most professionals and managers came from nursing homes (82.6%) with privately‐built and privately‐operated ownership.

**Table 3:** Sample characteristics (*N* = 37).

Characteristics	Workshop 1 (*n* = 25)	Workshop 2 (*n* = 15)	Total (*n* = 37)
Sex			
Female (*n*, %)	19 (76%)	12 (80%)	29 (78.4%)
Age, years			
Mean (range)	37 (20–59)	33 (23–46)	35 (20–59)
Professional role			
Healthcare professional			
Physician	2	/	2
Nurse	6	1	7
Social worker	5	5	9
Management			
Head nurse/nurse director	4	2	5
Director of facility	8	1	8
Academic in			
Palliative care	/	3	3
Other related fields	/	3	3
Experience, years			
Median (range)	7 (1–18)	2.5 (1–21)	5 (1–21)
Professional title			
None	6	6	12
Junior	6	1	6
Mid‐level	9	6	14
Senior	4	2	5
Employer			
Types of LTCFs			
Nursing homes	19	1	19
Residential care facilities	4	/	4
Ownership of LTCFs			
Government‐built and privately‐operated	4	/	4
Privately‐built and privately‐operated	19	1	19
NGO	1	4	4
Universities	/	6	6
Hospitals	1	4	4

Abbreviations: LTCFs, long‐term care facilities; NGO, non‐governmental organisation.

### 3.2 Findings of the ToC workshops

**Figure 2:**
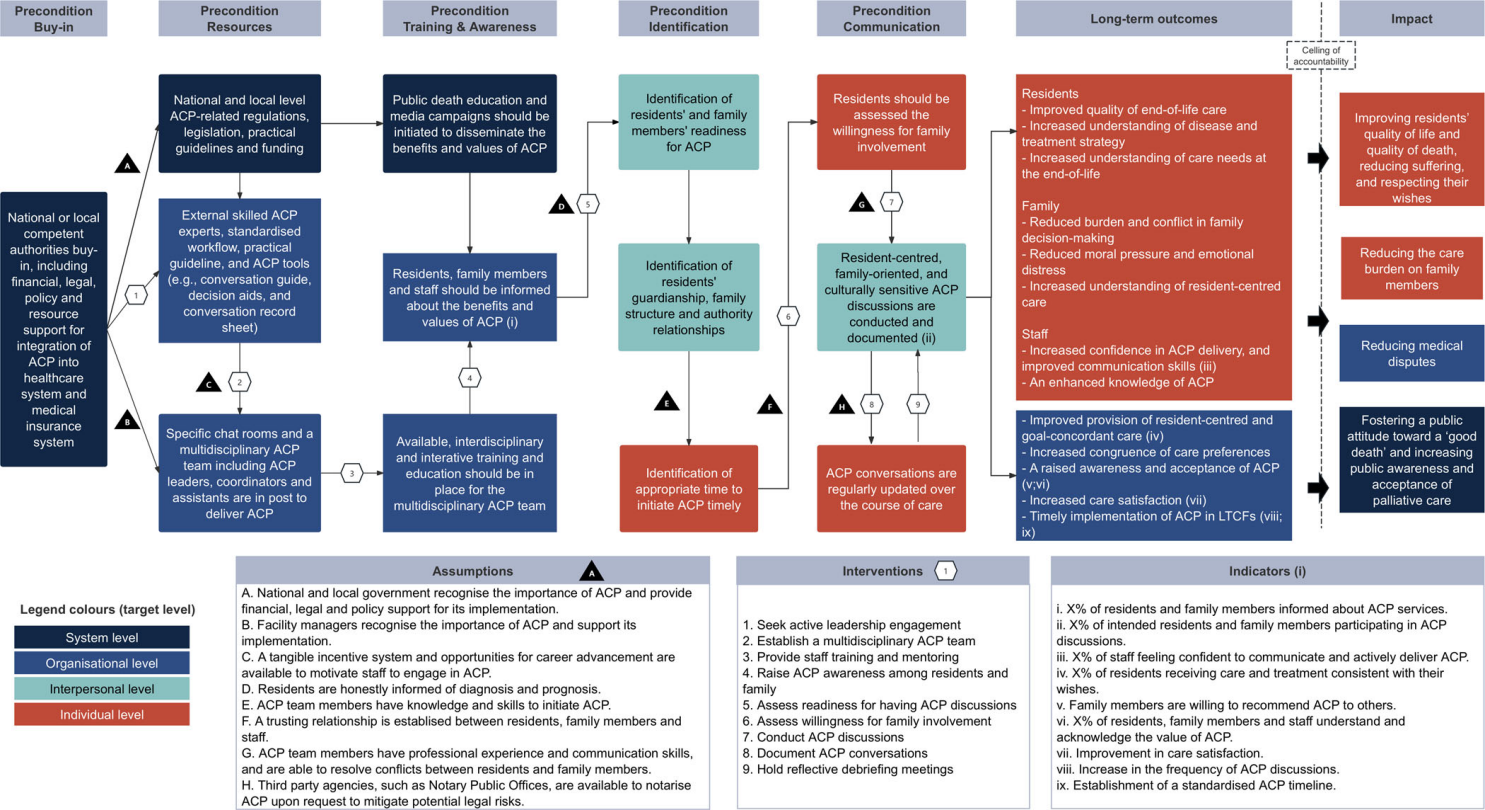
Programme theory depicting the implementation, processes and outcomes influencing ACP in Chinese LTCFs.

A programme theory is depicted in a ToC map (Figure 2), providing a visual representation of the causal pathways through which ACP is hypothesised to achieve impacts in Chinese LTCFs and outlining the key elements required to bring about change.

#### 3.2.1 Impact

The desired real‐world impacts of ACP were identified at individual, organisational and system levels. These included: improving residents' quality of life and quality of death, reducing suffering, respecting their wishes; reducing the care burden on family members; reducing medical disputes; fostering a public attitude toward a ‘good death’ (shifting the focus from overtreatment to dying with dignity); and increasing public awareness and acceptance of palliative care.

‘This [ACP] also leads to a shift in attitude. Traditional filial piety makes it difficult for people to accept (the idea of forgoing overtreatment). However, how can we raise the awareness (of dying with dignity)? Such effort should not only be made at the organisational level but also at the broader societal level.’

Group 3, Workshop 1

#### 3.2.2 Long‐Term Outcomes

The long‐term outcomes were identified at the individual and organisational levels. At individual levels, residents' long‐term outcomes were improved quality of end‐of‐life care, increased understanding of disease, treatment strategy and care needs at the end of life. For families, the long‐term outcomes were reduced burden and conflict in family decision‐making, reduced moral pressure and emotional distress, and increased understanding of resident‐centred care. Staff‐related long‐term outcomes included increased confidence in ACP delivery, improved communication skills and enhanced knowledge of ACP. At the organisational level, the long‐term outcomes included increased congruence of care preferences among residents, family members and staff, raised awareness and acceptance of ACP, improved resident‐centred and goal‐concordant care, increased care satisfaction and timely implementation of ACP in LTCFs.

#### 3.2.3 Preconditions

##### 3.2.3.1 Buy‐In

To achieve long‐term outcomes, all participants agreed that the first precondition in the pathway was government buy‐in from national or local competent authorities. This would provide financial, legal, policy and resource support for the integration of ACP into the existing healthcare and medical insurance systems. In addition, the government buy‐in would promote ACP implementation in LTCFs and reduce potential legal risks to staff and facilities.

‘If we truly want to promote ACP, a key issue is mitigating its legal risks. Ultimately, this comes down to the field of superstructure—how laws, regulations, and policies at both local and national levels can support its implementation. While there have been some grassroots efforts, progress has been limited. The main concern among many of us [healthcare professionals] is that it [implementing ACP] may lead to legal complications.’

Group 2, Workshop 1

##### 3.2.3.2 Resources

Participants then identified the resources needed to implement ACP in LTCFs, which were categorised at the system and organisational levels. At the system level, ACP‐related regulations, legislation, practical guidelines and funding should be available to promote ACP implementation. At the organisational level, participants emphasised the importance of the availability of internal and external programme resources. As staff in LTCFs often lack the knowledge and skills needed to conduct ACP, it was considered essential to have external skilled trainers or experts to supervise their practice. In addition, standardised workflow and practical guidelines would help to normalise ACP into routine practice. ACP tools such as conversation guides, decision aids and conversation record sheets to track the provision of ACP‐concordant care should also be provided with external support.

Internal programme resources included the availability of specific chat rooms and human resources to deliver ACP. Participants stressed the importance of a multidisciplinary ACP team with doctors, nurses, social workers and psychologists to promote holistic care. Participants noted the importance of defining ‘*Who is in charge of ACP?*’ Defined roles such as ACP leaders, coordinators and assistants with clear responsibilities were considered vital for interprofessional collaboration. Having a high staff turnover in LTCFs emphasised the importance of careful selection of ACP team members to sustain quality delivery.

‘In a multidisciplinary ACP team, the roles of leaders, coordinators, and supporting members should be clearly defined. Otherwise, in practice, things will be in a mess. Involving too many professionals without clear responsibilities can lead some members to become passive observers rather than active contributors. Potential power conflicts may also arise within the team.’

Group 1, Workshop 2

##### 3.2.3.3 Training and awareness

Participants felt that education and media campaigns should be initiated to increase public awareness of the benefits and values of ACP. This could be achieved through multimedia platforms, public service advertising, public welfare activities and the distribution of brochures in hospitals and communities. At the organisational level, residents, family members and staff need to be informed about ACP benefits. Participants highlighted the importance of using lay language and integrating ACP information into routine care (e.g., at admission, or through brochures, videos and bulletin boards).

Moreover, interdisciplinary training and education should be in place for the multidisciplinary ACP team to improve their skills and competency. This training should include medical knowledge, ethics, psychology and communication skills, along with the related policies and regulations, workflows, practical guidelines and ACP tools. Suggested training approaches included multidisciplinary discussions, interactive training sessions, external training activities and seminars. Participants also highlighted the need for iterative training based on real‐world practice.

##### 3.2.3.4 Identification

Participants noted that ACP may not be suitable for every resident and that identifying the right people and the right time to initiate ACP was considered to be a key precondition. Tools to assess the readiness of residents and their families for ACP should be available, which consider factors such as educational level, health conditions, mental capacity, psychological status, life experiences and cultural beliefs. In addition, participants highlighted the indispensability of family involvement. It would be crucial to identify residents' guardianship (when close relatives are authorised to make legally binding decisions for the residents), and family dynamics (e.g., structure and authority relationships) to ensure active family involvement.

‘It is important to consider the resident's family structure and relationships…. In the context of Chinese culture, when it comes to end‐of‐life treatment decisions, a guardian may not always be able to make decisions that fully represent the views of the entire family. Therefore, it is essential to first examine the complexity of family dynamics. If only the guardian is involved in the discussion, can they truly speak for the whole family?’

Group 3, Workshop 2

In terms of the right time, participants identified that ACP should be initiated as soon as possible, preferably in the early stages of admission. In addition, daily conversations that signal residents' or families' willingness to engage in ACP discussions or events, such as hospital transfers or group activities, may precipitate ACP discussions. Social workers and nurses were identified as suitable staff to initiate ACP conversations.

##### 3.2.3.5 Communication

Sometimes, residents may find it difficult to express their wishes in the presence of their families. To avoid this, residents' willingness to involve families in ACP discussions and the appropriate approach (e.g., joint or separate discussion) should be assessed. The ACP discussions should be resident‐centred and family‐oriented, that is, focus on respecting residents' wishes while also considering the emotional, psychological and bereavement needs of the family. In addition, discussions should be culturally and religiously sensitive to residents' beliefs. Finally, the ACP conversations should be recorded to monitor the provision of goal‐concordant care and should be regularly updated.

‘Additionally, if the resident and their guardian discuss this topic together, can the resident truly express their thoughts? Should we communicate separately with the resident, then with the family, before bringing them together for a joint discussion?’

Group 3, Workshop 2

#### 3.2.4 Interventions

Nine intervention components were identified to enable one precondition to logically move to the next: (1) seeking active leadership engagement; (2) establishing a multidisciplinary ACP team; (3) providing staff training and mentoring; (4) raising ACP awareness among residents and family; (5) assessing readiness for having ACP discussions; (6) assessing willingness for family involvement; (7) conducting ACP discussions; (8) documenting ACP conversations and (9) holding reflective debriefing meetings.

#### 3.2.5 Indicators

Participants identified nine indicators for evaluating the achievement of outcomes in ACP implementation: (1) the proportion of residents and family members informed about ACP services; (2) the proportion of intended residents and family members participating in ACP discussions; (3) the proportion of staff feeling confident to communicate and actively deliver ACP; (4) the proportion of residents receiving care and treatment consistent with their wishes; (5) family members are willing to recommend ACP to others; (6) the proportion of residents, family members and staff understand and acknowledge the value of ACP; (7) improvement in care satisfaction; (8) increase in the frequency of ACP discussions and (9) establishment of a standardised ACP timeline. Participants also highlighted that, given the expected survival time of residents in LTCFs, the endpoint for evaluation should be 6 months or until the residents' deaths.

#### 3.2.6 Assumptions

Eight external conditions should be in place to achieve outcome pathways: (1) national and local government recognise the importance of ACP and provide financial, legal and policy support for its implementation; (2) facility managers recognise the importance of ACP and support its implementation; (3) a tangible incentive system and opportunities for career advancement are available to motivate staff to engage in ACP; (4) residents are honestly informed of diagnosis and prognosis; (5) ACP team members have knowledge and skills to initiate ACP; (6) a trusting relationship is established between residents, family members and staff; (7) ACP team members have professional experience and communication skills and are able to resolve conflicts between residents and family members; and (8) third party agencies, such as Notary Public Offices, are available to notarise ACP upon request to mitigate potential legal risks.

## 4 Discussion

This is the first known study to use the ToC approach to develop complex palliative care interventions in China. It generated a culturally and contextually appropriate programme theory that outlines the causal pathways through which the ACP is hypothesised to achieve impacts in Chinese LTCFs, with a focus on understanding its implementation, processes and outcomes (Figure 2).

Our findings resonate with existing literature on the importance of family involvement in ACP implementation, particularly in the East Asian contexts, where they play a central role in decision‐making and information gatekeeping [41, 42, 43]. Family involvement is especially important in LTCFs, where many older people experience fluctuating cognitive trajectories and require the timely appointment of surrogate decision‐makers [44]. Compared to those developed in a Western context [20], our ACP programme theory clearly demonstrates the mechanisms to ensure family integration and identifies key family‐related intervention components. These include raising family awareness of ACP, assessing residents' willingness for family involvement, conducting family‐oriented ACP discussions and addressing the needs of family members. These components are likely transferable to the Asian contexts and align with a recent international consensus on ACP in dementia, which recommends supporting both individuals and their families from the outset, ensuring that decisions can be made together [44]. Previous studies have also suggested that healthcare professionals should avoid assumptions and assess what family involvement means to individuals and their relatives to create a safe environment where residents feel comfortable expressing their true feelings and preferences [44, 45]. Assessing family dynamics is a valuable approach for identifying family roles, relationships, communication styles, patterns of interaction and factors that shape these interactions in routine practice [46].

A vital precondition for ACP implementation in LTCFs was the establishment of a multidisciplinary team with clearly defined roles and responsibilities. The challenge of ascertaining who is in charge of ACP within institutions has been identified in previous studies, with ACP discussions often not taking place within a multidisciplinary team due to role confusion [14, 47]. Physicians hold primary responsibility for the medical aspects of ACP, including sharing information on diagnosis and prognosis, initiating and withdrawing medical treatments, and addressing ethical and legal considerations [48]. Meanwhile, professionals from other disciplines, such as social workers and nurses, often establish closer relationships with residents and their families and bring psychosocial considerations into the conversations [24]. Our findings suggested that allocating healthcare professionals with specific roles (e.g., leaders, coordinators and assistants) and providing interdisciplinary training might help establish a sense of ownership and enable better interdisciplinary collaboration and communication [49].

This study highlighted the potential impacts of ACP at the systems level in terms of fostering public attitudes towards a ‘good death’ and increasing public awareness and acceptance of palliative care. This echoes the significant cultural challenges faced by palliative care in China and other Asian countries, such as low public awareness, overtreatment and information concealment due to filial piety, and cultural taboos surrounding death [19, 50, 51]. ACP can be framed as a window of opportunity for public health education and awareness campaigns, especially for older people [52]. Upstreaming ACP conversations and normalising them into life milestones can help reduce the stigma around discussing death, dying, loss and caring, while iteratively enhancing individuals' palliative care awareness and death literacy [52, 53].

### 4.1 Implications for Future Practice and Policy

Our mid‐range programme theory can serve as a heuristic tool that is adaptable for context‐specific ACP interventions in other countries. Along with the five preconditions necessary for achieving desired ACP outcomes, the study identified ways to promote tailored and effective family involvement. Our programme theory highlights implementation considerations specific to LTCFs, such as the availability of external resources, high staff turnover and inadequate staff training. A combination of the bottom‐up approach, wherein ACP is promoted with organisational leadership buy‐in, and supportive top‐down policies and legislation is critical for establishing an ACP culture and ensuring normalised implementation. The outcomes and indicators identified can be used to guide the selection of outcome measures in future evaluation studies.

### 4.2 Strengths and Limitations

Our programme theory adopted a ‘systems thinking’ approach to understanding how ACP can be integrated into health services socioecologically rather than focusing on a single sector [20, 40]. Online delivery of ToC workshops is a novel approach. From our experience, online ToC is particularly appropriate for running international studies that involve cross‐country workshops and a diverse population [54]. Using collaborative online whiteboard platforms can mimic the drawing process of constructing ToC maps during workshops.

However, the Covid‐19 pandemic restrictions limited the involvement of residents and family members in the ToC workshops. In addition, many residents and families had very limited lived experience with ACP [55]. To address this, the findings of interviews with residents and family members, as well as vignettes developed based on interview cases, were presented to participants [14]. PPI consultation also ensured that the views of residents and family members were considered when refining the programme theory. Secondly, most participants came from nursing homes with privately built ownership, which may have limited the generalisability of results to some extent. Thirdly, whilst performed in other ToC studies [56, 57], we did not rank the identified outcomes and indicators due to time and resource constraints. This somewhat limited our understanding of the importance of the primary and secondary outcomes that an ACP is expected to achieve. Furthermore, the intervention components identified were focused on the individual and organisational levels. It is important that system‐level ACP interventions, such as providing public death education and establishing ACP referral networks, are also considered to promote ACP from a public health perspective. Finally, although ToC approach has been increasingly used to inform the design of palliative care interventions [56, 58], there has been insufficient evidence to demonstrate whether this approach leads to significantly more effective interventions [28]. In subsequent research, we will test and refine the programme theory in terms of its feasibility and acceptability in Chinese LTCFs.

## 5 Conclusion

This study contributed to a culturally and contextually appropriate programme theory that outlines the causal pathways through which the ACP is hypothesised to achieve impacts in Chinese LTCFs, with a focus on understanding its implementation, processes and outcomes. The programme theory highlights the complexity of family dynamics and identifies intervention components for family‐integrated ACP. It also underscores the critical role of supportive top‐down policies and other enabling elements, such as external resources and the multidisciplinary approach, in enhancing ACP implementation in LTCFs.

## Author Contributions

**Yuxin Zhou:** conceptualisation, methodology, investigation, formal analysis, data curation, writing ‐ original draft, writing – review and editing, project administration. **Ariel Wang:** validation, formal analysis, writing – review and editing. **Clare Ellis‐Smith:** conceptualisation, formal analysis, writing – review and editing, supervision. **Debbie Braybrook:** conceptualisation, formal analysis, writing – review and editing, supervision. **Haixia Feng:** validation, investigation, writing – review and editing. **Richard Harding:** conceptualisation, formal analysis, writing – review and editing, supervision.

## Ethics Statement

This study was approved by King's College London Research Ethics Committee (Ref: HR/DP‐20/21‐23549) and the Independent Ethics Committee for Clinical Research of Zhongda Hospital, Affiliated to Southeast University (Ref: 2021ZDSYLL277‐P01).

## Consent

Informed consent was obtained from all participants before the workshops.

## Conflicts of Interest

The authors declare no conflicts of interest.

## Supporting information

4‐Supplematary Material.

## Data Availability

The datasets used and/or analysed during the current study are available from the corresponding author upon reasonable request.
