# Supplementary material for: Implementation, Processes and Outcomes of Advance Care Planning: A Culturally and Contextually Appropriate Programme Theory Developed in Chinese Long‐Term Care Facilities
Source: Health Expect. 2025 May 8;28(3):e70291. doi: 10.1111/hex.70291 (PMC12061843; doi:10.1111/hex.70291)
Supplement: Supplementary file 1 — 4‐Supplematary Material. [file HEX-28-e70291-s001.docx]

**Supplementary Material 1. Reporting checklists**

**Checklist for reporting Theory of Change (ToC) in Public Health Interventions**

| **Item** | **Guide questions/description** | | **Reported on Page No.** |
| --- | --- | --- | --- |
| *1. Is the ToC approach defined?* | | | |
| a | Is a definition of ToC given by the authors? | | 3 |
| b | Do the authors explain their reasons for using a ToC approach? | | 3 |
| *2. Is the ToC development process described?* | | | |
| a | Are the methods used to develop the ToC, such as stakeholder meetings and interviews, document reviews, programme observation, existing conceptual frameworks or published research, described? | | 3;5-7 |
| b | Where stakeholders are involved, is it clear how many stakeholders participated, what their role is in relation to the intervention, how they were consulted (e.g., number of interviews, focus groups, ToC workshops) and the extent to which the consultations were participatory? | | 5-7;9-10 |
| c | Is the method used to compile the data into a ToC described? (including how disagreements between stakeholders were resolved) | | 5-7 |
| d | Is the extent to which stakeholders were able to validate the resultant ToC and were owners of the final product described? | | 5-7 |
| *3. Is the resultant ToC (or a summary thereof) depicted in a diagrammatic form and does it include?* | | | |
| a | The long-term outcome or impact of the intervention. | 12 | |
| b | The anticipated short and medium term outcomes and the process of change. | 12-15 | |
| c | The intervention components which happen at different stages of the pathway. | 15; Figure 2 | |
| d | The context of the intervention. | Was explored in previous primary qualitative study | |
| e | Assumptions about how change would occur. | 15-16; Figure 2 | |
| f | Additional ToC elements such as indicators, supporting research evidence, beneficiaries, actors in the context, sphere of influence and timelines where relevant. | 15; Figure 2 | |
| *4. Is the process of intervention development from the ToC described* | | | |
| a | Are the methods of how interventions were refined from the ToC to something which can be implemented described? (For example, further stakeholder workshops, interviews, systematic literature reviews) | | N/A |
| *5. Is the way in which the ToC was used to develop and implement the evaluation described?* | | | |
| a | Are evaluation research questions generated from the ToC? | | N/A |
| b | Is the role of ToC in the design, plan or conduct of the evaluation clear? | | N/A |
| c | Does the paper describe the extent to which the key elements described in the ToC were measured in the evaluation (i.e. impact, short and medium term outcomes and the process of change, context, assumptions and the intervention)? | | N/A |
| d | Does the paper describe whether and how process indicators were used to improve the quality of the intervention? | | N/A |
| e | Is the role of the ToC in the analysis of the results of the evaluation clear? | | N/A |
| f | Is the role of ToC in the interpretation of the results of the evaluation described? (including the breakdown of programme theory, unanticipated outcomes and causation including the strength and direction of causal relationships) | | N/A |

Developed from: Breuer, E., Lee, L., De Silva, M., & Lund, C. (2015). Using theory of change to design and evaluate public health interventions: a systematic review. *Implementation Science*, *11*(1), 1-17.

**Consolidated criteria for reporting qualitative studies (COREQ): 32-item checklist**

| **Topic** | **Item No.** | **Guide questions/description** | **Reported on Page No.** |
| --- | --- | --- | --- |
| **Domain 1: Research team and reﬂexivity** | | | |
| *Personal Characteristics* | | | |
| Interviewer/facilitator | 1 | Which author/s conducted the interview or focus group? | 5-6 |
| Credentials | 2 | What were the researcher’s credentials? E.g. PhD, MD | 5 |
| Occupation | 3 | What was their occupation at the time of the study? | 5 |
| Gender | 4 | Was the researcher male or female? | 5 |
| Experience and training | 5 | What experience or training did the researcher have? | 5 |
| *Relationship with participants* | | | |
| Relationship established | 6 | Was a relationship established prior to study commencement? | 5 |
| Participant knowledge of the interviewer | 7 | What did the participants know about the researcher? e.g. personal goals, reasons for doing the research | 5 |
| Interviewer characteristics | 8 | What characteristics were reported about the interviewer/facilitator? e.g. Bias, assumptions, reasons and interests in the research topic | 5-7 |
| **Domain 2: study design** | | | |
| *Theoretical framework* | | | |
| Methodological orientation and Theory | 9 | What methodological orientation was stated to underpin the study? e.g. grounded theory, discourse analysis, ethnography, phenomenology, content analysis | 3-4 |
| *Participant selection* | | | |
| Sampling | 10 | How were participants selected? e.g. purposive, convenience, consecutive, snowball | 5 |
| Method of approach | 11 | How were participants approached? e.g. face-to-face, telephone, mail, email | 5 |
| Sample size | 12 | How many participants were in the study? | 9-10 |
| Non-participation | 13 | How many people refused to participate or dropped out? Reasons? | 9 |
| *Setting* | | | |
| Setting of data collection | 14 | Where was the data collected? e.g. home, clinic, workplace | 5 |
| Presence of non-participants | 15 | Was anyone else present besides the participants and researchers? | No |
| Description of sample | 16 | What are the important characteristics of the sample? e.g. demographic data, date | 9-10 |
| *Data collection* | | | |
| Interview guide | 17 | Were questions, prompts, guides provided by the authors? Was it pilot tested? | 5-6 |
| Repeat interviews | 18 | Were repeat interviews carried out? If yes, how many? | N/A |
| Audio/visual recording | 19 | Did the research use audio or visual recording to collect the data? | 5 |
| Field notes | 20 | Were ﬁeld notes made during and/or after the interview or focus group? | 5-7 |
| Duration | 21 | What was the duration of the interviews or focus group? | 9 |
| Data saturation | 22 | Was data saturation discussed? | 5 |
| Transcripts returned | 23 | Were transcripts returned to participants for comment and/or correction? | No |
| **Domain 3: analysis and findings** | | | |
| *Data analysis* | | | |
| Number of data coders | 24 | How many data coders coded the data? | 7 |
| Description of the coding tree | 25 | Did authors provide a description of the coding tree? | 7 |
| Derivation of themes | 26 | Were themes identiﬁed in advance or derived from the data? | 7 |
| Software | 27 | What software, if applicable, was used to manage the data? | 7 |
| Participant checking | 28 | Did participants provide feedback on the ﬁndings? | Figure 1 |
| *Reporting* | | | |
| Quotations presented | 29 | Were participant quotations presented to illustrate the themes/ﬁndings? Was each quotation identiﬁed? e.g. participant number | 11-16 |
| Data and ﬁndings consistent | 30 | Was there consistency between the data presented and the ﬁndings? | 11-16 |
| Clarity of major themes | 31 | Were major themes clearly presented in the ﬁndings? | 11-16 |
| Clarity of minor themes | 32 | Is there a description of diverse cases or discussion of minor themes? | 11-16 |

Developed from: Tong A, Sainsbury P, Craig J. Consolidated criteria for reporting qualitative research (COREQ): a 32-item checklist for interviews and focus groups. *International Journal for Quality in Health Care*. 2007. Volume 19, Number 6: pp. 349 – 35

**Supplementary Material 2. Terminology for Theory of Change in this study**

| **Terminology** | **Definition** |
| --- | --- |
| Impact | The ultimate real-world change the intervention trying to affect. The intervention may contribute towards achieving this impact but cannot achieve it solely on its own. |
| Ceiling of accountability | The level after which the intervention is not accountable for the outcomes on its own. Line is often drawn between long-term outcomes and impact. |
| Long-term outcome | The changes that the intervention is directly accountable for. This will be the primary and secondary outcomes of the evaluation. |
| Precondition | The intended outcomes of the intervention need to be realised for the long-term outcomes to be achieved. |
| Intervention | The different components of the complex intervention. These represent the actions or activities that need to be undertaken to bring about a specific intermediate outcome. |
| Assumption | An external condition beyond the control of the intervention must exist for a precondition to be achieved (e.g., policy support). |
| Indicator | Things that can be measured to determine whether have achieved intermediate outcomes. |

Reference

1. De Silva MJ and Lee L. Using theory of change in the development, implementation and evaluation of complex health interventions. A practical guide. [https://www.mhinnovation.net/sites/default/files/downloads/resource/MHIN%20ToC%20guidelines_May_2015_0.pdf](https://www.mhinnovation.net/sites/default/files/downloads/resource/MHIN%20ToC%20guidelines_May_2015_0.pdfn)
2. De Silva, M. J., Breuer, E., Lee, L., Asher, L., Chowdhary, N., Lund, C., & Patel, V. (2014). Theory of change: a theory-driven approach to enhance the Medical Research Council's framework for complex interventions. Trials, 15(1), 267.

**Supplementary Material 3. Examples of event artefacts generated during two workshops**

**
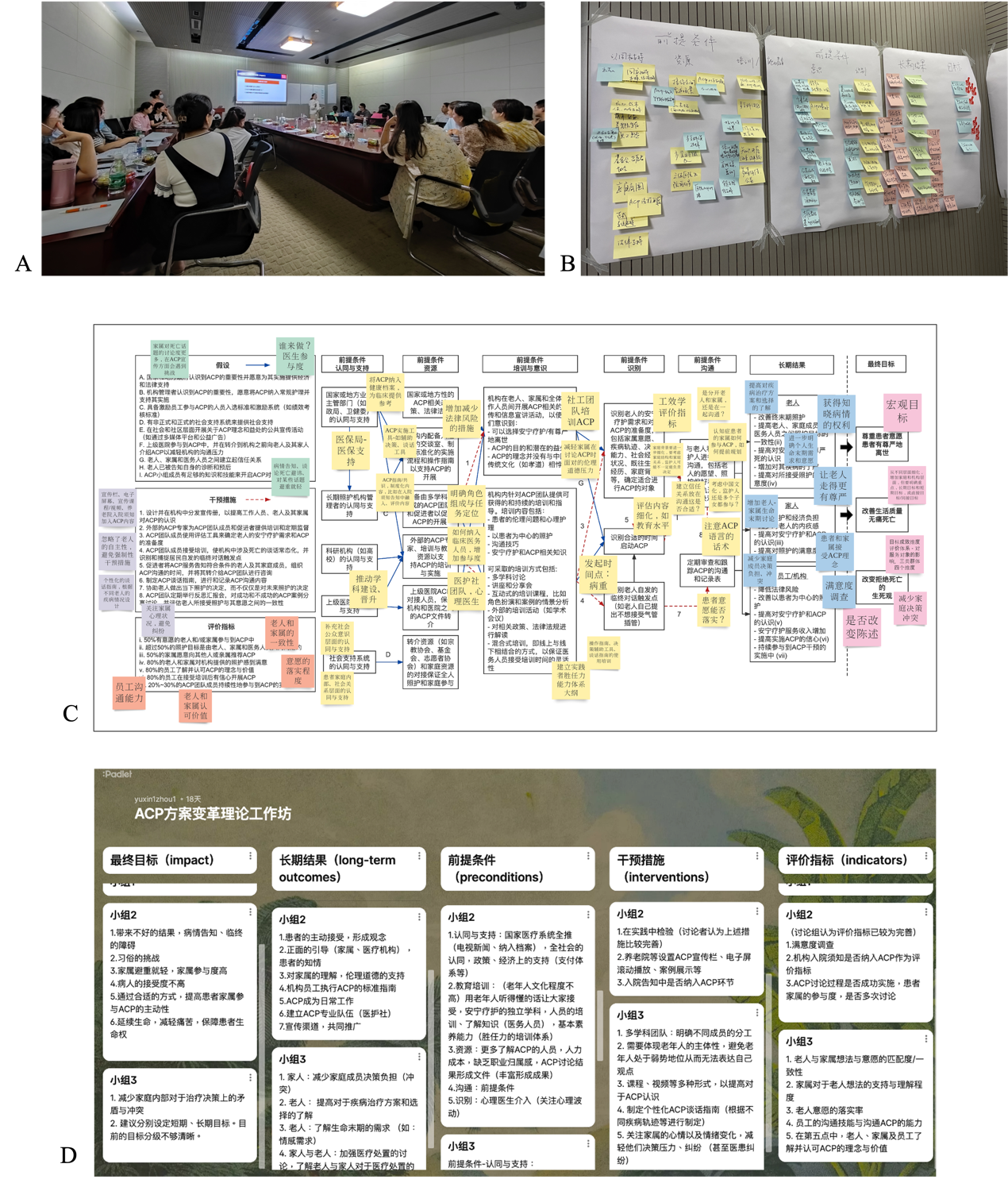
**

Note: Examples of event artefacts include photographs of in-person workshop activities (A), Theory of Change maps generated during two workshops (B, C) and Padlet field notes scribed during the online workshop (D).
